# Clinical Insights Regarding the Targeted Chromosomal Region for Mosaicism and Aneuploidy in Embryos in IVF Treatment and Literature Review

**DOI:** 10.3390/diagnostics15111375

**Published:** 2025-05-29

**Authors:** Bogdan Doroftei, Alexandra Savuca, Nicoleta Anton, Radu Maftei, Ana-Maria Cretu, Anca Roxana Bivoleanu, Mara Doroftei, Ciprian Ilea

**Affiliations:** 1 Origyn Fertility Center, Palace Street No. 3C, 700032 Iasi, Romania; bogdandoroftei@gmail.com (B.D.); dr.radu.maftei@gmail.com (R.M.); dabuleanu.ana@gmail.com (A.-M.C.); 2 Department of Mother and Child Care, University of Medicine and Pharmacy “Grigore T. Popa”, University Street No. 16, 700115 Iasi, Romania; a.bivoleanu@yahoo.com (A.R.B.); cilea1979@yahoo.com (C.I.); 3 Clinical Hospital of Obstetrics and Gynecology “Cuza Voda”, Cuza Voda Street No. 34, 700038 Iasi, Romania; 4 Department of Obstetrics and Gynecology, Faculty of Medicine, University of Medicine and Pharmacy “Grigore T. Popa”, University Street No. 16, 700115 Iasi, Romania; 5 Ophthalmology Department, Faculty of Medicine, University of Medicine and Pharmacy “Grigore T. Popa”, University Street No. 16, 700115 Iasi, Romania; anton.nicoleta1@umfiasi.ro; 6 Ophthalmology Clinic, Sf. Spiridon Emergency Clinical Hospital of Iasi, University Street No. 16, 700111 Iasi, Romania; 7 Faculty of Medicine, University of Medicine and Pharmacy “Grigore T. Popa”, University Street No. 16, 700115 Iasi, Romania; maradoro06@gmail.com

**Keywords:** mosaicism, IVF, aneuploidy, PGT-A, chromosomal region

## Abstract

**Background**: Given the common occurrence of mosaicism and aneuploidy in IVF embryos, our study aimed to retrospectively identify whether specific chromosomal regions or individual chromosomes are predominantly affected in our clinic. Understanding these patterns can improve embryo selection, reduce miscarriage risks, and enhance genetic counseling. At the same time, due to the limited data on potential comorbidities in affected children, our findings aim to support both clinicians and patients in making informed decisions. **Methods**: The retrospective clinical study included 461 PGT-A biopsies from our clinic database (September 2023–December 2024) to determine whether specific chromosome regions or individual chromosomes (C) are more likely to be mosaic or aneuploid. **Results**: Among the 461 embryos analyzed in our clinic, the incidence rate of mosaicism was 16.70% whereas the aneuploidy rate was 32.10%. Our results showed that mosaicism tends to target a specific chromosomal region in embryos, namely the chromosome 1 to 9 region, in particular chromosomes 7, 1, 9. On the other hand, aneuploidy targets the chromosomal region chromosome 16 to 22, particularly chromosomes 16, 19, and 22. **Conclusions**: Our data suggest that mosaicism and aneuploidy affect the genome in an uneven manner and are often concentrated in specific chromosomal regions, with mosaicism primarily affecting the C1–C9 region and aneuploidy targeting the C16–C22 region. These data highlight the need for further research to understand these patterns and the impact of IVF methods on chromosomal targeting. Comparative studies could also be helpful in genetic counseling by clarifying the implications of the levels of mosaicism in the newborn.

## 1. Introduction

Embryonic development into a healthy neonate is a complex and well-regulated process that requires precise gene expression over time. Errors in early development can be the cause of implantation problems [1]. Aneuploidy is predominantly lethal to the embryo and/or the fetus and increases the risk of pregnancy loss dependent on the number of protein-coding genes affected, particularly in the context of trisomies 16 and 22 [2,3]. The main generating mechanisms of mosaic aneuploidies are represented by the postzygotic mitotic chromosome segregation errors and postzygotic mitotic monosomy/trisomy recovery of a pre-existing aneuploidy of meiotic origin. Clinical implications of chromosomal mosaicism are directly related to the type of characteristic. Some of these characteristics include the size, gene content, and number of copies. Others include the distribution of abnormal cells in different tissues and the ratio of normal to abnormal cells. It is important to educate patients throughout genetic counseling about the associated risks of each applicable type of chromosomal abnormality, as well as the odds of having a live birth [4].

The incidence of mosaicism detected by the next-generation sequencing (NGS) at the blastocyst stage varies considerably between clinics, with values ranging from 2% to 40%, with most centers reporting a frequency between 5% and 15%, depending on the analyzed age group [5,6]. Recent research suggests that embryos with complete or segmental mosaicism can lead to viable pregnancies, but such cases are associated with a lower implantation rate and a higher risk of miscarriage, or both phenomena occurring simultaneously [7,8]. Although the success rate of mosaic embryo transfer may be comparable to that of euploid embryo transfer, a >40% success rate of live births has been reported [9]. However, when comparing embryos with low mosaicism to euploid embryos, there was a 15.1% reduction in pregnancy progression up to 14 weeks [10].

Prenatal diagnosis of aneuploidy is very important because this condition is one of the main causes of death even in the uterine phase [11]. In addition, monosomies/trisomies are involved in several serious syndromes; even those with a longer life expectancy do not have the same quality of life as an euploid patient [12]. Overall, all autosomal monosomies and most trisomies are lethal at the embryonic stage, except for trisomies 13, 18, and 21, which cause Patau, Edwards, and Down syndromes, respectively. The fact that patients with Down’s syndrome can survive into adulthood suggests that it may not be a matter of viability per se, but rather an effect on development [13].

Over time, several methods have been introduced to analyze aneuploidy, such as fluorescence in situ hybridization (FISH) used to detect aneuploidies of chromosomes (C) 13, 18, 21, X, and Y in uncultured amniotic fluid cells or chorionic villi, using DNA samples [14]. From the blastocyst stage, the 23 chromosome sets can be verified using the next-generation sequencing (NGS) techniques, namely preimplantation genetic testing for aneuploidy (PGT-A) [15].

Although it is not desirable, in cases where there are no euploid embryos to transfer, the Preimplantation Genetic Diagnosis International Society (PGDIS) suggests that mosaic trisomies 1, 3, 4, 5, 6, 8, 9, 10, 11, 12, 17, 19, 20, 22, X, and Y are preferable to transfer. In contrast, the mosaic trisomies 2, 7, 13, 14, 15, 16, 18, and 21 are disregarded in the case of transfer. It should also be noted that confined placental mosaicism of certain chromosomes (especially 2, 7, 16, and possibly 22) may be associated with an increased risk of intrauterine growth restriction and other pregnancy complications, including fetal demise [16].

Moreover, a recent study has indicated that pregnant women who are diagnosed with confined placental mosaicism during pregnancy face an elevated risk of adverse pregnancy outcomes, including impaired fetal growth and hypertensive disorders in the mother. This observation underscores the necessity for enhanced antenatal surveillance and monitoring [17].

Counseling and interpreting these genetic tests remain challenging. This is due to the lack of information about transferring mosaic embryos and guidelines.

However, any segmental aneuploidy could theoretically allow live birth in the presence of a euploid cell line, and the resulting phenotype will likely depend on the proportion of abnormal cells and the type of tissue involved [16]. 

Broadly speaking, mosaicism and aneuploidy are common chromosomal abnormalities in IVF embryos. These can affect both the success of implantation and the outcome of pregnancy. Certain chromosomal regions may be more susceptible than others, and the distribution of these abnormalities at the chromosomal level is not fully understood.

Identifying these regions is essential to optimize embryo selection and to counsel patients about the chances of successful embryo transfer. For clinicians, a better understanding of mosaicism and aneuploidy patterns can help refine diagnostic and embryo selection protocols, reducing the rate of failed implantation and the risk of miscarriage. It can also help to minimize the uncertainty associated with prognosis and guide decisions about whether to recommend mosaic embryo transfer.

Moreover, understanding not only the likelihood of a successful pregnancy but also the potential medical implications for the child is essential for patients. Currently, the literature provides limited data on comorbidities associated with specific mosaicisms and aneuploidies, making accurate counseling of prospective parents difficult.

Another aspect that should be noted is regarding race and ethnicity. For instance, in the United States (US), a seven-year analysis of PGT-A use among minority women was conducted. The findings suggest that, despite a substantial increase in the overall utilization of PGT-A during the period 2014–2020, its application remained consistently lower in ART cycles for black and Hispanic women compared to white women. Conversely, there was a substantial increase in the utilization of PGT-A among Asian women during this period [18]. The role of socio-demographic factors in this context had been explored also in US. The authors posit that a racial and ethnic differentiation exists with regard to the utilization of PGT. Consequently, Hispanic patients and those who do not speak English have significantly lower access to PGT analyses compared to other racial/ethnic groups. The necessity of patient education was emphasized, along with the importance of overcoming language barriers between healthcare providers and patients to enhance the access to medical care [19]. Regarding the associations with IVF outcomes, findings indicated that late loss of pregnancy, preterm birth, live birth, term birth, and stillbirth were found to be related to ethnicity. Also, substantial disparity was identified between Hispanic and non-Hispanic ethnicities [20]. These concerns regarding the discrepancies in pregnancy outcomes after IVF have been attributed by researchers to potential differences in genetic susceptibility. Researchers have indicated the potential for ethnic variations in aneuploidy rates, which could contribute to inequalities in IVF success from the United Kingdom, India and Japan data. Consequently, this raises concerns about the adequacy of standardized protocols. Furthermore, given the heterogeneous nature of ethnic groups, the existence of significant genetic, cultural, and environmental diversity within a group cannot be excluded. This increases the complexity of the issue, making it more difficult to elucidate [21]. In addition, the relationship between race and embryonic mosaicism remains to be elucidated. The findings from another study do not support a significant relationship between race and embryonic mosaicism; however, a possibility for low-level mosaicism was identified in a US study [22].

The available data indicate the presence of a persistent knowledge gap that merits attention, particularly in light of the growing number of clinical real-world data from multiple global centers, especially in the European region, taking into account the substantial heterogeneity between ethnic groups.

Therefore, the aim of this study was to retrospectively analyze our PGT-A database in order to identify the most frequently affected chromosomes by mosaicism and aneuploidy in our IVF center; to improve embryo selection strategies and to better inform patients about the possible health risks of their future newborn.

In addition, this study aims to contribute to the specialized literature with data from a Caucasian population in an Eastern European center where these services are not as accessible as in other geographical areas of the world.

## 2. Materials and Methods

Our clinic’s database of preimplantation genetic testing for aneuploidy (PGT-A), which analyzes chromosomal constitution, was used as the basis for our clinical study. A total number of 461 biopsies performed between September 2023 and December 2024 were included in our retrospective study. Biopsy protocols, laboratory personnel, embryologists, specimen handling protocols, kits, internal algorithms and sequencing analysis software was not changed throughout the period.

### 2.1. Embryo Biopsy and PGT-A Analysis

Embryo biopsies were performed according to ESHRE guidelines and recommendations [23]. The zona pellucida (ZP) was opened using a 400–500 µs laser pulse (Lykos Laser, Hamilton Thorne Biosciences, Beverly, MA, USA) on the fourth day of embryonic development, only when necessary. Embryos at the blastocyst stage (days 5, 6, and 7 of development) were biopsied. The biopsy was performed under a Leica DM6000B inverted microscope (Wetzlar, Germany). The microscope was equipped with a Leica AM6000 micromanipulator (Hamilton Thorne Biosciences, Beverly, MA, USA) and a Lykos laser (Hamilton Thorne Biosciences, Beverly, MA, USA). The embryo, with the inner cell mass visible and away from the ZP opening, was fixed in the desired position with the support pipette. Trophectoderm cells were slowly aspirated into the biopsy pipette and gently collected by applying negative pressure. While the blastocyst remained attached to the support pipette, 5Wetzlar10 cells (but no more than 10) were left in the lumen of the pipette. Tubing of the biopsied cells was performed using sterile materials, gown, mask, cap, and gloves. Nuclease-Free Water + PBS was used for washing the biopsied cells then transferred to PCR tubes containing 2.5 µL medium. After this step, centrifugation was performed for 1 min at 2000 rpm (Spermfuge SF 800, Fornax, Mumbai, India) and stored in the freezer.

Molecular analyses were performed by our in-house laboratory, Origen. DNA samples were subjected to next-generation sequencing (NGS) using the EmbryoMap™ Sample Prep Kit (Vitrolife, Gothenburg, Sweden) for PGT-A analysis on the Illumina MiSeq platform (Illumina, San Diego, CA, USA) according to the manufacturer’s protocols and guidelines. To analyze sequencing data and report chromosomal abnormalities was used the eMap software v1.0.1 (Vitrolife, Gothenburg, Sweden). Samples with a mosaicism of less than 20% were classified as euploid, and those with values greater than 80% were classified as aneuploid.

### 2.2. Inclusion Criteria

For aneuploid embryos, only embryos with no more than two affected chromosomes were included. In the case of mosaic embryos, the embryos included in the study were those with a mosaicism rate between 20% and more than 50% and were categorized as follows: Low Mosaic (20–30%); Medium Mosaic (30–50%); and High Mosaic (>50%).

### 2.3. Statistical Analysis

One-way ANOVA analysis using Graph Prism Pad 9 software (San Diego, CA, USA) was used to compare differences between chromosomal regions. A *p*-value < 0.05 was considered to be significant.

## 3. Results

Euploidy was found in 38.61% cases, aneuploidy in 32.1%, mosaicism in 16.7%, and undiagnosed in 12.58% of the 461 total embryos analyzed in our database (Table 1).

### 3.1. Distribution of Affected Chromosomes by Mosaicism Class

Among all embryos diagnosed with mosaicism in our clinic, the Low Mosaic category was the most prevalent, accounting for 49% of cases. This was followed by the Medium Mosaic category, representing 39%, while the High Mosaic category was the least frequent, occurring in 12% of cases.

The distribution of the number of the affected chromosomes across mosaicism classes reveals distinct patterns. In the Low Mosaic class (15–30%), which is the most frequent of the three categories, the number of affected chromosomes varies considerably. Higher frequencies have been identified for several chromosomes (chromosome 1 (n = 7), chromosome 7 (n = 8), chromosome 9 (n = 5)). In contrast, no frequencies were observed for chromosome 5 and chromosome 10.

The prevalence of the medium mosaic class (30–50%) presents higher frequencies on chromosomes 4 (n = 7), 9 (n = 5) and 5 (n = 4), while chromosomes 12, 16, and 17 remained unaffected, suggesting that certain chromosomes may be more prone to moderate mosaicism.

**Figure 1:**
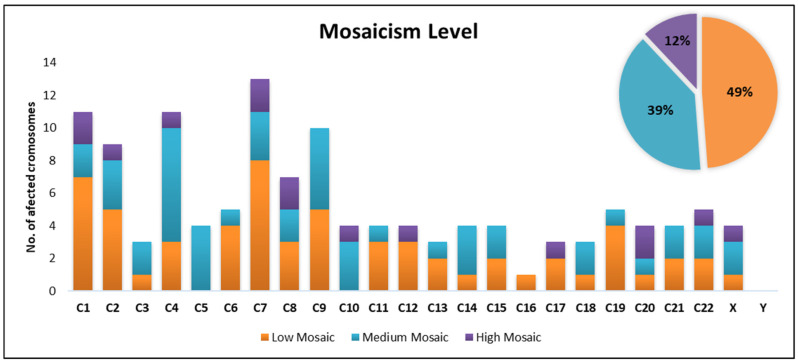
Distribution of affected chromosomes by mosaicism classes.

In contrast, the prevalence of the high mosaic class (>50%) is significantly lower compared to the other two classes, with many values of 0 or 1 for most chromosomes. Few chromosomes (1, 7, 8, 20) showed higher values (n = 2). Overall, this group displayed a more uniform pattern, characterized by a markedly lower number of affected chromosomes (Figure 1).

Chromosome 7 appears to be the most severely affected, exhibiting high values across all categories, followed by chromosome 1, 4, and 9. Chromosomes 4, 5, and 9 showed elevated values especially for medium mosaic category, which could suggest a predisposition to mosaicism at this intermediate level (Figure 1).

**Figure 2:**
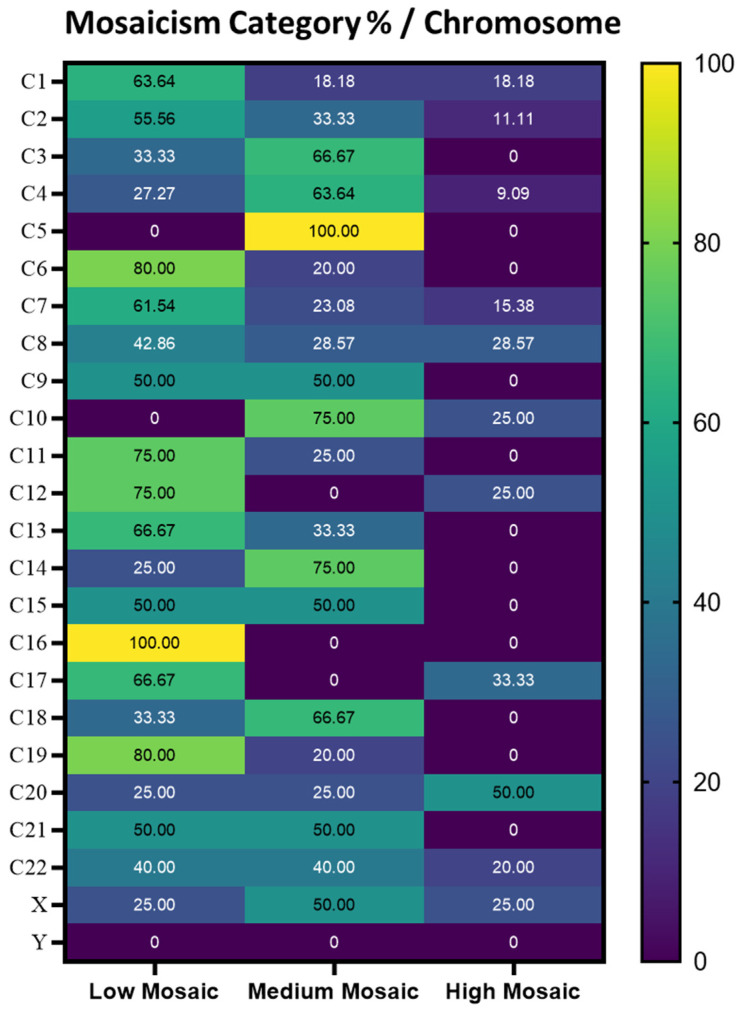
Heatmap representing the percentage of each class of mosaicism in each chromosome.

Furthermore, when referring to the percentage distribution of mosaicism categories per chromosome, chromosome 5 had a proportion of 100% for the Medium Mosaic category and chromosome 16 had proportion of 100% for the Low Mosaicism category. Additionally, chromosomes 6 and 19 showed a strong association with Low Mosaicism, with 80% of cases falling into this category (Figure 2).

In consequence, the Low Mosaic class is predominant on chromosomes 1, 2, 6, 7, 8, 11, 12, 13, 17, and 19, and it is exclusively present on chromosome 16, while the Medium Mosaic class is more prevalent on chromosomes 3, 4, 10, 14, and 18, with chromosome 5 being the only chromosome where it appears exclusively.

The High Mosaic class is primarily observed on chromosome 20, though its distribution remains minimal. Additionally, chromosomes 21 and 22 exhibit an equal distribution between the Low and Medium Mosaic classes.

### 3.2. Chromosomal Region Differentiation

**Figure 3:**
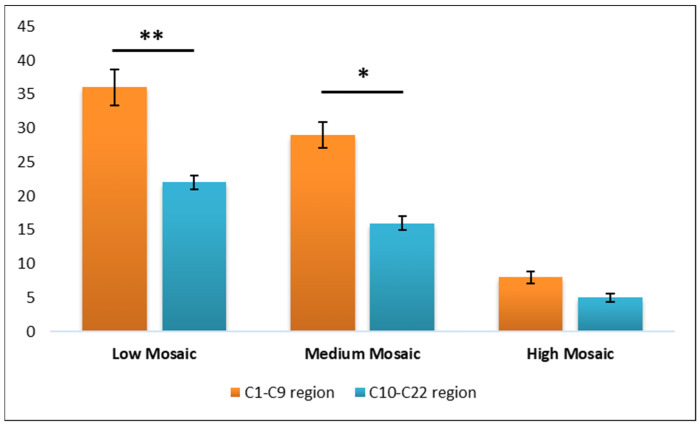
Graphical representation of the chromosomal region’s differentiation. Data are presented as mean ± ST. DEV. A *p* value < 0.05 was considered statistically significant and denoted * and a *p* value < 0.001 was denoted **.

Given the predominance of affected values in the chromosome 1 to 9 region, we further analyzed the mean values for each mosaicism class by comparing the C1–C9 and the C10–C22 regions. This analysis revealed a statistically significant difference between the two regions for both the Low Mosaic class (*p* = 0.003) and the Medium Mosaic class (*p* = 0.012), suggesting a distinct distribution pattern of mosaicism across these chromosomal regions (Figure 3).

### 3.3. The Percentage of Chromosomes Affected and the Average Mosaicism Rate

**Figure 4:**
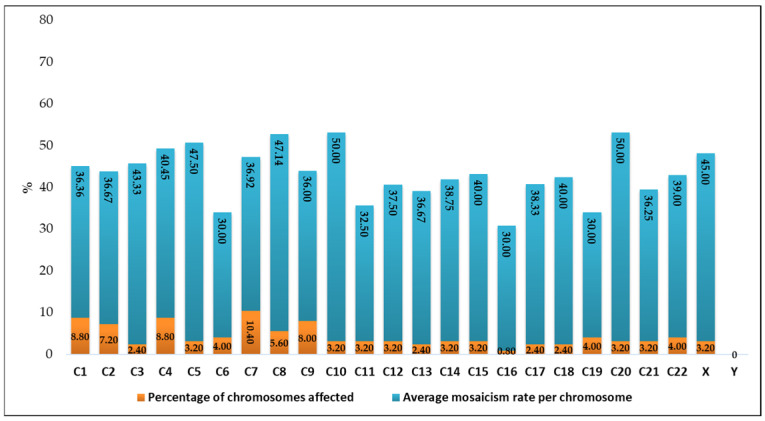
Graphical representation of the percentage of chromosomes affected and the average mosaicism rate per chromosome.

The chromosomes most frequently identified with mosaicism were chromosome 7 (n = 13, 10.40%) with a mean mosaicism rate of 36.92%, followed by chromosome 4 (n = 11, 8.8%) with 40.45%, chromosome 1 (n = 11, 8.8%) with 36.36%, and chromosome 9 (n = 10, 8%) with 36%. At the opposite end of the spectrum, chromosome 16 (n = 1, 0.8%) exhibited a mean mosaicism rate of 30%, while chromosome Y (n = 0, 0%) showed no detected mosaicism (Figure 4).

The chromosomes most affected in terms of average mosaicism rate (severity) were chromosome 10 (n = 4, 3.2%) with a mean mosaicism rate of 50%, followed by chromosome 20 (n = 4, 3.2%) at 47.5%, chromosome 8 (n = 7, 5.6%) at 46.14%, chromosome X (n = 4, 3.2%) at 45%, and chromosome 3 (n = 3, 2.4%) at 43.33%. At the opposite end of the spectrum, chromosome 6 (n = 5, 4%), chromosome 16 (n = 1, 0.8%), and chromosome 9 (n = 5, 8%) all exhibited a mean mosaicism rate of 30%, while chromosome Y (n = 0, 0%) showed no detected mosaicism.

Considering both frequency and severity, chromosome Y and chromosome 16 appear to be the least affected by mosaicism (Figure 4).

### 3.4. The Percentage of Chromosomes Affected by Mosaicism for Each Embryo Analyzed

**Figure 5:**
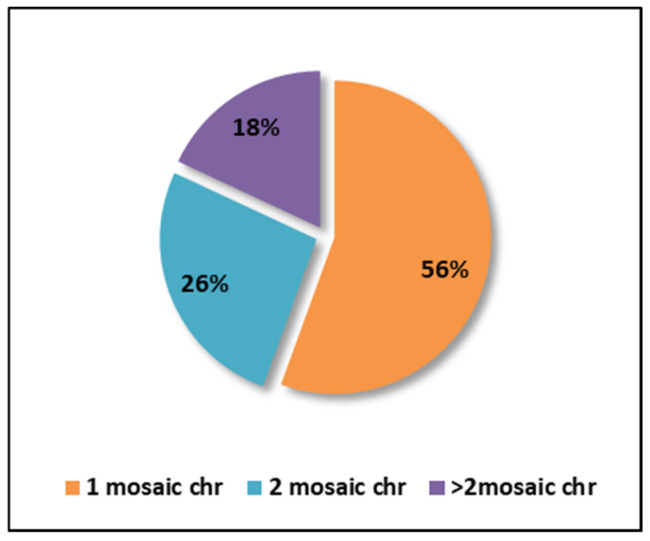
Graphical representation of the percentage of chromosomes affected by mosaicism (chr = chromosomes).

Regarding the percentage of chromosomes affected by mosaicism per embryo, the analysis of the 72 embryos diagnosed with mosaicism revealed that more than half (56%) had only one affected chromosome. Additionally, 26% of embryos exhibited two affected chromosomes, while the remaining 18% had three to five affected chromosomes (Figure 5).

### 3.5. Aneuploidy

**Figure 6:**
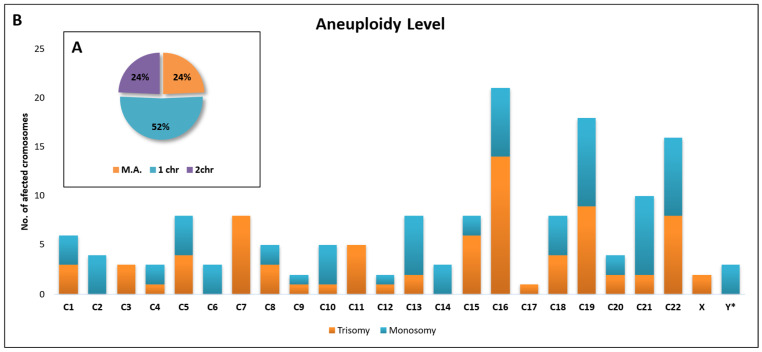
Graphical representation of the percentage of chromosomes affected by aneuploidy (**A**) and their distribution (**B**).

Out of a total of 148 embryos diagnosed with aneuploidy, slightly more than half (52%) had only one deletion or addition on a single chromosome. Additionally, 24% of embryos had two affected chromosomes, while another 24% exhibited three or more affected chromosomes (multiple aneuploidy—M.A.) (Figure 6A). The only embryos with one or two chromosomes were included in this analysis regarding the distribution of the number of affected chromosomes (n = 148).

In terms of trisomy, most cases were identified for chromosome 16, followed by chromosomes 19 and 22 with the same number of affected embryos as chromosome 7.

For monosomy, most cases were identified for chromosome 19, followed by chromosomes 21 and 22 with an equal number of cases, followed by chromosome 16.

In general, chromosomes 16, 19, 22, and 21 were the most affected chromosomes by aneuploidy. Three cases of monosomy X (denoted Y* on the graph) and two cases of trisomy XXY (denoted X on the graph) were identified (Figure 6B).

## 4. Discussion

A primary aspect to discuss regarding PGT-A analysis is the technology used to perform it. Studies in the specialized literature use both array and NGS technology. Trophectoderm biopsy along with NGS technology for PGT analysis is an efficient strategy to identify the suitable embryos for transfer [24]. Research findings indicate that the prevalence of mosaicism is notably higher in the NGS-based PGT group compared to the SNP array–based PGT group. Moreover, the total mosaicism detection rate with NGS was found to be significantly higher than that with SNP array-based PGT (23.3% vs. 7.7%) [25]. In addition, NGS technology has been shown to exhibit both efficiency and robustness when compared with aCGH methods. The use of NGS for the detection of euploid blastocysts for transfer has been shown to result in pregnancy rates that are comparable to those observed with aCGH screening (74.7% vs. 69.2%). Additionally, the observed implantation rates were comparable between the NGS and aCGH groups (70.5% vs. 66.2%) [26]. Lately, third-generation sequencing (Nanopore) gained interest in PGT-A analysis. A study showed that it could represent a viable alternative to current commercial NGS-based PGT-A solutions for aneuploidy and segmental imbalance in trophectoderm biopsy samples [27]. Our data and the studies chosen to discuss are exclusively analyzed using NGS technology.

Our data showed that the incidence of mosaicism in the analyzed embryos was 16.70%, while the rate of aneuploidy was 32.10%. A review by Leight et al., analyzing published clinical data from 2021, found that the incidence of mosaicism at the blastocyst stage using the NGS techniques is variable among centers, with reports ranging from 2 to 40%. However, most clinics report a mosaicism rate between 5 and 15%, depending on the age group investigated [6]. In another study, which was performed on a sample of 5718 blastocysts, the rate of mosaicism was 21.8% (1245 blastocysts). Of these, 60.2% were classified as low-grade mosaicism with 20–49% of abnormal cells and 39.8% were classified as high-grade mosaicism with 50–80% of abnormal cells. In addition, the authors mentioned that the rate of mosaicism was significantly higher in embryos biopsied on day 6 than in those biopsied on day 5, and that it increased in an inverse manner with the morphological score [28]. Coll et al., state in their manuscript that the overall frequency of mosaicism identified in their study was 13.9% on a sample of 1708 blastocysts also analyzed using NGS technology [29]. In another study, the incidence of mosaicism ranged from 11% to 25.7% of the embryos examined, with an average of 18.6%. The authors note that for these embryos, the mosaic status was known prior to transfer in 83.6% of cases. In 16.4% of cases, the embryo was transferred under the assumption of euploidy, but re-evaluation of the sequencing profile after transfer resulted in the embryo being assigned to the mosaic category [30].

As far as the number of chromosomes affected by mosaicism is concerned, our data showed that 26% of the embryos tested had two chromosomes affected and 18% had between three and five chromosomes affected. Thus, embryos with >3 affected chromosomes would have a reduced pregnancy potential compared to embryos with only one or two affected chromosomes. Even though no significant difference was observed in this regard between mosaicism involving one versus two chromosomes [31]. Likewise, Coll et al. 2021, showed a prevalence of 11.8% cases of two chromosomes affected by mosaicism along with 6.3% in ≥3 chromosomes [29]. In addition, a significant correlation between the number of affected chromosomes and unfavorable outcomes has been highlighted [30].

Our data indicate a predilection for mosaicism in the chromosome 1 to 9 region. This predilection was also reported in another study where the frequency of single segment aneuploidy (SSA) was identified in two thirds of the blastocysts analyzed in the same region. The remainder (29.6%) of aneuploidies were on autosomes and sex chromosomes. In addition, the authors hypothesize that the segmented acrocentric chromosomes (21, 22, and Y) could present a lower rate of segmental aneuploidy in their data, similar to the present study, due to the limits of detection caused by the small size of these chromosomes [32].

In the specialized literature, X chromosome mosaicism has been identified quite frequently in women using ART techniques. However, although it is not extremely frequent in our results, X chromosome mosaicism is mainly in the “medium mosaic” category (50%). Studies showed that, this mosaicism can be generated by proliferating cells in culture and is closely related to the advanced age of the woman [33]. However, mosaicism due to culture conditions can also be relatively distinguished by involving a limited number of cells and aneuploidy of several chromosomes, not only the X chromosome.

Nevertheless, the true mosaicism and clinical significance are not fully understood [34]. In addition, X chromosome abnormalities are usually associated with abnormal sexual development and amenorrhea, infertility, recurrent abortions, and premature ovarian failure [35]. Segmental copy number variations can lead to different syndromes and conditions, e.g., the terminal deletion on the p-arm of chromosome 5 causes Cri-du-chat syndrome, and Wolf–Hirschhorn syndrome is also generated by a partial deletion on the short arm of chromosome 4 [36].

In our data, chromosomes 4 and 5 showed elevated values especially for the medium mosaic category. Notably, chromosome 5 is the only one without any cases of low mosaic and only 4 cases of medium mosaic, which makes it atypical. A similar situation is observed in chromosome 16, which present exclusively low mosaic cases. Another chromosome predominantly affected by mosaicism in our data is chromosome 9, similar to the Escriba’s study, along with chromosomes 4 and 1 and predominantly in the chromosome 1 to 9 region [32]. These findings raise the possibility that mosaicism may predominately be in the chromosome 1 to 9 region.

Moreover, mosaicism on chromosome 7 was the most prevalent in our data. An analysis of published cases with chromosome 7 mosaicism mentions that in the prenatal phase, the most identified phenotype would be facial asymmetry and developmental delay. In the postnatal period cases with pigmentary changes in the skin, hypotonia, short palpebral fissure, sparse hair, and facial asymmetry have been reported [37]. In a 2021’s study, the chromosomes most affected by mosaicism were 1, 5, and 9. Almost similar when considering that in our study the most affected chromosomes were chromosomes 7, 9, and 1. Authors also suggest that chromosomes 1 and 9, due to their size, may be more susceptible to breakage leading to segmental anomalies. In addition, the abundance of regions with heterochromatin blocks can lead to breaks. These regions are difficult for DNA replication and may also be a cause [29].

The fact that maternal meiotic errors are age-dependent is an interesting aspect to discuss. For example, in women over 35 years of age, studies have shown an increase in aneuploidies for all chromosomes. Chromosomes 3, 15, 16, 18, 19, 21, and 22 showed a disproportionate increase at older ages. Given that these chromosomes are associated with the rate of implantation and are also commonly involved in clinically recognized abnormal pregnancies, this suggests that the clinical risk of aneuploidy increases with age [38]. At the blastocyst stage, the distribution of aneuploidies among the 23 sets of chromosomes is generally uneven. In a study of 5000 embryos, the most frequent aneuploidies were found in the relatively small chromosomes, namely 15, 16, 21, and 22. Chromosomes 1 and 6 were the least affected, with relatively equal proportions of monosomies and trisomies [13]. Our results are in agreement with these, where the most affected chromosomes are 16, 19, 21, and 22. Similarly, a predominance of trisomy and monosomy was found on chromosomes 15, 16, 21, and 22 in an Italian study [39].

In our data, trisomy 16 was the most prevalent; similarly, in a clinical study from Denmark, the presence of placental trisomy 16 was detected in 25/49 cases. In 16% of these cases, adverse pregnancy outcomes were observed, ranging from small gestational age (SGA) to fetal malformations and intrauterine death, confirming the trend toward an association with a high frequency of trisomy 16 cells [40]. In another study conducted in the United States, of forty-four families with mosaic trisomy and limited placental mosaicism detected prenatally for trisomy 16, 68.2% of the infants were female and most susceptible to the disease. The most frequent pregnancy complications were gestational hypertension (38.1%), preterm delivery (71.4%), cesarean section (73.8%), birth weight < 10th percentile (73.8%), neonatal intensive care unit (NICU) admission (88.1%), and congenital anomaly (59.5%). In contrast, 81.8% of the school-aged children were fully enrolled in regular classes, and the mean physical, psychosocial, and total HRQOL scores were high: 90.6 (34.4–100), 86.7 (35–100), and 84.8 (34.8–100), respectively, where 100 represents optimal quality of life. Body asymmetry was described in 21.4% of the children. Congenital cardiac, genitourinary, and musculoskeletal anomalies were also present [41].

Trisomy 19, the second most common in our data, is rare in neonates, with many cases known to end in spontaneous abortion in the first trimester [42]. Studies have shown that the average life expectancy is 0.8 months, even if the child is born without proper medication [43]. However, small face, dysmorphic features (e.g., short neck and stature, downturned corners of mouth, abnormal ears) along with seizures, intellectual disability, and motor development and speech delay have been observed in patient with this trisomy [44]. On the other hand, monosomy of chromosome 19, the most prevalent monosomy in our data, is associated with pre/postnatal growth retardation, psychomotor/language delay, hyperactivity, brachydactyly, hearing loss, anteverted nares, short neck, and hypodontia [45].

Trisomy 22, which is also a frequent occurrence in our data, is often associated with spontaneous abortion. Microcephaly, broad flat nasal bridge, epicanthic folds, ocular hypertelorism, microtia, palatal clefts, webbed neck, heart and kidney defects, hypoplastic distal phalanges, and genital defects in males are commonly characterized by trisomy 22 in fetuses, along with intrauterine growth restriction [46]. In the case of monosomy 21, which was prevalent in our data, the literature mentions that it has been rarely observed. Most probably, monosomy 21 is lethal from the uterine phase, and cases of spontaneous abortion have been reported in fetuses with this condition [47]. Furthermore, certain phenotypes, such as a wide nasal bridge, prominent epicanthic folds, simple ears, but also bilateral overlapping fifth fingers, were observed in a newborn born with 45% mosaicism of chromosome 21 in the region 21q11.2q22.13, characteristics also found in patients with Down syndrome. The case study mentioned that death was pronounced at the age of 4.5 months, which highlights the need for detailed studies, but also for early detection, starting from the neonatal phase [48].

Overall, regions of chromosomes 6, 7, 11, 14, 15, and 20 are associated with known imprinting disorders. Analysis of these regions should be considered prior to implantation by using PGT analysis [49].

Moreover, in case of sex chromosomes, these aneuploidies can have different clinical consequences, from undetectable to lethal, depending on the number of copies and the chromosome involved. For example, while cases 47, XXX and 47, XYY usually lead to phenotypically normal females and males, cases 45, X and 47, XXY lead to Turner’s and Klinefelter’s syndromes, but the X chromosome is absolutely essential because its absence invariably leads to embryonic death [13].

One of the current dilemmas is the implantation or prioritization of embryos with a certain degree of mosaicism. Depending on the type of chromosome involved, one recommendation would be to transfer some embryos with mosaicism. Thus, mosaicism involving chromosomes 14 and 15 was discouraged due to the perceived risk of uniparental disomy. In addition, abnormalities of chromosomes 2, 7, and 16 are associated with intrauterine growth retardation, and chromosomes 13, 18, and 21 are considered problematic.

In general, recommendations for the transfer of embryos with mosaicism remain cautious in the absence of fully conclusive data; therefore, a conservative approach to transfer is appropriate [5]. Recent studies have shown that embryos with segmental mosaicism can lead to pregnancies. However, it has been suggested that such transfers may also be associated with an increased risk of miscarriage. The live birth rate (LBR) is quite positive for both low and high mosaicism (44.5% vs. 36%), but the risk of miscarriage is higher for embryos with high mosaicism (30.7% vs. 5.1% for low mosaicism) [7]. In another prospective double-blind study, no cases of mosaicism or uniparental disomy were detected in pregnancies or neonates from low and medium mosaic embryos. In addition, obstetric and neonatal outcomes were similar between the study groups, with embryos having the same developmental potential as euploid embryos [50]. However, in another study, mosaic embryos showed inferior clinical outcomes compared with euploid embryos. Although the percentage of aneuploid cells did not correlate with the outcome, the type of mosaicism did. Embryos with single mosaic segmental aneuploidies showed better outcomes than all other types. Furthermore, the study supports the fact that mosaic blastocysts from oocytes retrieved at maternal ages <34 have better outcomes than those retrieved at older maternal ages [8].

Another aspect that should be considered is the risks associated with embryo biopsy, as has been recently highlighted by researchers. Although long-term adverse effects are uncommon, their potential existence is a valid concern. Presently, there is a paucity of comprehensive data, especially concerning the neurological spectrum, encompassing mental, cognitive, and psychomotor outcomes, along with blood pressure and anthropogenic outcomes in the post-childhood period. However, researchers have identified the potential for adverse outcomes, including lower birth rate, abnormal placentation, hypertensive disorders of pregnancy, preterm delivery, or birth defects [51].

Furthermore, it is noteworthy that the biopsy technique itself may also influence the targeting of a particular chromosomal region for abnormalities. This perspective warrants further examination and research in the future. In addition, to ensure the quality of ART procedures without ethnic barriers and current knowledge gaps, it is imperative to consider data and research on more ethnic categories. Collecting data from different centers is essential for this scientific endeavor.

### Limitations of the Study

We acknowledge that this is a small-scale study with data from a single center, but we consider that these findings are very valuable and that the investigation of this hypothesis needs to be extended by more studies in different clinics, with the present study still being a pioneer in this field.

## 5. Conclusions

Our findings indicate that mosaicism and aneuploidy do not affect the genome uniformly but tend to concentrate in distinct chromosomal regions, with mosaicism primarily affecting the chromosomes 1 to 9 region and aneuploidy affecting the chromosomes 16 to 22 region. The possibility that certain protocols (e.g., biopsy technique) may influence this genetic response, both in our data and in the literature, suggests potential underlying biological mechanisms that warrant further investigation. Additionally, understanding these patterns could enhance IVF embryo selection strategies and genetic counseling by providing more precise risk assessments. Future research should explore the potential impact of IVF methodologies on chromosomal instability and refine guidelines for evaluating mosaic embryos, along with a better genetic counseling of patients with regard to the implications of certain levels of mosaicism in a newborn.

## Figures and Tables

**Table 1:** Percentage of diagnoses identified in the analyzed embryos.

Euploid	Aneuploid	Mosaic	No Diagnosis
38.61%	32.10%	16.70%	12.58%

## Data Availability

Data are contained within the article.

## Abbreviations

IVF	in vitro fertilization
PGT-A	preimplantation genetic testing for aneuploidy
C	chromosome
NGS	next-generation sequencing
FISH	fluorescence In Situ hybridization
PGDIS	Preimplantation Genetic Diagnosis International Society
ZP	zona pellucida
ART	assisted reproductive technologies
NICU	neonatal intensive care unit
M.A.	multiple aneuploidy
